# Effect of the mining pipeline on habitat quality and the diversity of semiaquatic bug communities (Heteroptera: Gerromorpha) in streams of the eastern Amazon

**DOI:** 10.1007/s10661-026-15086-7

**Published:** 2026-03-05

**Authors:** Lucas Nogueira Laurindo, Beatriz Luz-Silva, Fábio Santos-Silva, Ingrid Reis Campos, Joás Silva Brito, Karina Dias da Silva, Bethânia Oliveira de Resende, Gabrielly Silva Melo, Alana Patricia Meguy Guterres, Erlane José Cunha, Thaisa Sala Michelan, Luciano Fogaça de Assis Montag, Leandro Juen

**Affiliations:** 1 Laboratório de Ecologia E Conservação (LABECO), Instituto de Ciências Biológicas (ICB), Universidade Federal Do Pará, UFPA, Belém, Pará Brazil; 2 Programa de Pós-Graduação Em Ecologia PPGECO, Instituto de Ciências Biológicas, Universidade Federal Do Pará, UFPA, Belém, Pará Brazil; 3 School of Environmental Science, Simon Fraser University, Burnaby, BC Canada; 4 Programa de Pós-Graduação Em Ecologia E Evolução, Instituto de Ciências Biológicas, Universidade Federal de Goiás, UFG, Goiás, Goiânia Brazil; 5 Laboratório de Ecologia de Produtores Primários (ECOPRO), Instituto de Ciências Biológicas, Universidade Federal Do Pará, Rua Augusto Corrêa, Programa de Pós-Graduação Em Ecologia, Belém, PA 66075-110 Brazil; 6 Instituto Tecnológico Vale, Belém, Pará Brazil; 7 Museu Paraense Emílio Goeldi–Campus de Pesquisa, Belém, Pará Brazil

**Keywords:** Environmental impact, Conservation, Bioindicators, Biomonitoring, Hemiptera

## Abstract

Assessing how the disturbances affect the physical structure and biodiversity of freshwater ecosystems has become a critical research priority. Bauxite mining is economically important in the Amazon, but associated infrastructure may influence freshwater biodiversity. We evaluated the effects of a bauxite ore pipeline on the community structure of semiaquatic bugs (Heteroptera: Gerromorpha) in 40 streams of the eastern Amazon, including 20 sites crossed by the pipeline and 20 control sites. We tested whether (i) species abundance and richness upstream and downstream of control sites would be higher than in pipeline sites, (ii) upstream sites would show greater abundance and richness due to species sensitivity and habitat alteration in sites crossed by the pipeline, (iii) community composition would differ between pipeline and control sites, with characteristic species for each treatment, and (iv) the pipeline would indirectly influence communities through changes in habitat quality, riparian cover, and abiotic variables. Multivariate analyses indicated clear differences in community composition, with indicator species characterizing each condition, including the species *Rhagovelia jubata* in pipeline streams and *Stridulivelia alia* and *Stridulivelia strigosa* in control streams. Abundance showed a tendency to be higher in control streams, whereas richness was associated with abundance but not with habitat integrity. These findings indicate that, although diversity metrics remained similar, pipeline installation is linked to changes in species composition, reflecting shifts in local environmental conditions.

**Supplementary Information:**

The online version contains supplementary material available at 10.1007/s10661-026-15086-7.

## Introduction

Exploratory activities in the Amazon, such as deforestation and mining, have intensified in recent years in order to meet the growing human demand for raw materials (Allan, 2004; Barlow et al., 2016). As a consequence, the water bodies of this region become highly vulnerable to such exploitation and begin to lose their physical and structural characteristics (Cruz et al., 2025), as well as their aquatic species diversity (Wang et al., 2021; dos Santos et al., 2023). These changes lead to the homogenization of environments and the loss of species functionality (Chase et al., 2020), compromising the integrity and natural balance of freshwater ecosystems (Brasil et al., 2020a, b). Therefore, understanding how different sources of impact affect these communities is essential to indicate mitigation strategies for potential alterations and to support environmental management actions for decision-makers (Sundar et al., 2020).

Iron and bauxite mining has been widespread in the Amazon for over 50 years, contributing to economic development but also affecting freshwater ecosystems (Brito et al., 2021; Sonter et al., 2017). Bauxite, one of the world’s most economically important minerals, occurs in high concentrations in tropical regions, and about 85% of Brazil’s production is concentrated in the Amazon (Monteiro et al., 2017). A critical stage of this activity is ore transport, which in the region often requires moving material over long distances. Truck transport demands extensive road networks, vegetation removal, and soil disturbance, generating additional environmental impacts (Silva et al., 2024). As an alternative, companies have adopted underground pipelines connecting mines to refineries, which reduce some terrestrial impacts but still require vegetation clearing for access roads and infrastructure. These constructions may alter stream channels, disrupt drainage continuity, and promote local impoundment, potentially modifying aquatic habitats (Sonter et al., 2017).

In this context, the use of aquatic insects for environmental health assessment is essential not only for the monitoring of freshwater ecosystems but also because of the important ecological role these species play (Ferreira et al., 2024; Santos-Silva et al., 2025). Assessing the richness, abundance, and composition of these communities provides us with responses about environmental changes related to water quality and human activities (Merritt et al., 2017; Santos-Silva et al., 2025). These metrics allow monitoring of changes in the structure of biological communities, highlighting imbalances caused by alterations in environmental conditions, such as the removal of riparian vegetation (Dias-Silva et al., 2020). For companies, this information provides a scientific basis for the implementation of mitigation measures that minimize negative impacts on biodiversity, ensuring that their activities are sustainable and in compliance with legal and environmental requirements (Guerry et al., 2015; Nash et al., 2020).

Semiaquatic insects, particularly those of the infraorder Gerromorpha, are highly sensitive to environmental changes due to their close association with the water surface, dependence on riparian vegetation, and strong reliability on habitat integrity at the air–water interface (Cunha et al., 2015; Guterres et al., 2021) and have increasingly been employed in the monitoring of freshwater ecosystems (Barros et al., 2020; Santos-Silva et al., 2025). These insects are related to the physicochemical characteristic of streams (Dias-Silva., et al. 2020). Thus, more heterogeneous and less altered environments tend to exhibit higher diversity of aquatic insects (Cunha et al., 2015). This group displays unique physiological and morphological adaptations (Andersen and Weir 2004), and its species are predators adapted to life on the surface of water bodies (Andersen and Weir 2004), exhibiting morphological traits such as elongated bodies and legs (Santos et al., 2017), as well as hydrophobic hairs that aid in flotation. By preying on larvae of disease-transmitting mosquitoes, they contribute to ecosystem balance. In addition, Gerromorpha can be identified to species level, providing finer taxonomic resolution and increasing the sensitivity of ecological assessments, which, together with their ecological role and high sensitivity to disturbances, makes them effective bioindicators for assessing environmental impacts (Guterres et al., 2020; Dias-Silva et al., 2020).

Thus, the objective of this study is to evaluate the effect of pipeline implementation on the community of semiaquatic bugs (Heteroptera, Gerromorpha), using this group as a bioindicator to detect potential ecological alterations in Amazonian streams. For comparison, we used streams near the pipeline that show a certain level of conservation for the region. With this, we tested the following hypotheses: (i) species abundance and richness will be greater between upstream and downstream sections of control streams compared to pipeline streams; (ii) in pipeline streams, species abundance and richness will be greater in upstream sections than in downstream sections, due to species sensitivity and habitat alterations caused by the pipeline structure; (iii) community composition differs between areas under the influence of the pipeline and control areas, with specific species for each treatment; (iv) the pipeline structure has an indirect effect on the community mediated by changes in habitat quality, riparian cover, and limnological variables.

## Material and methods

### Study area

**Fig. 1:**
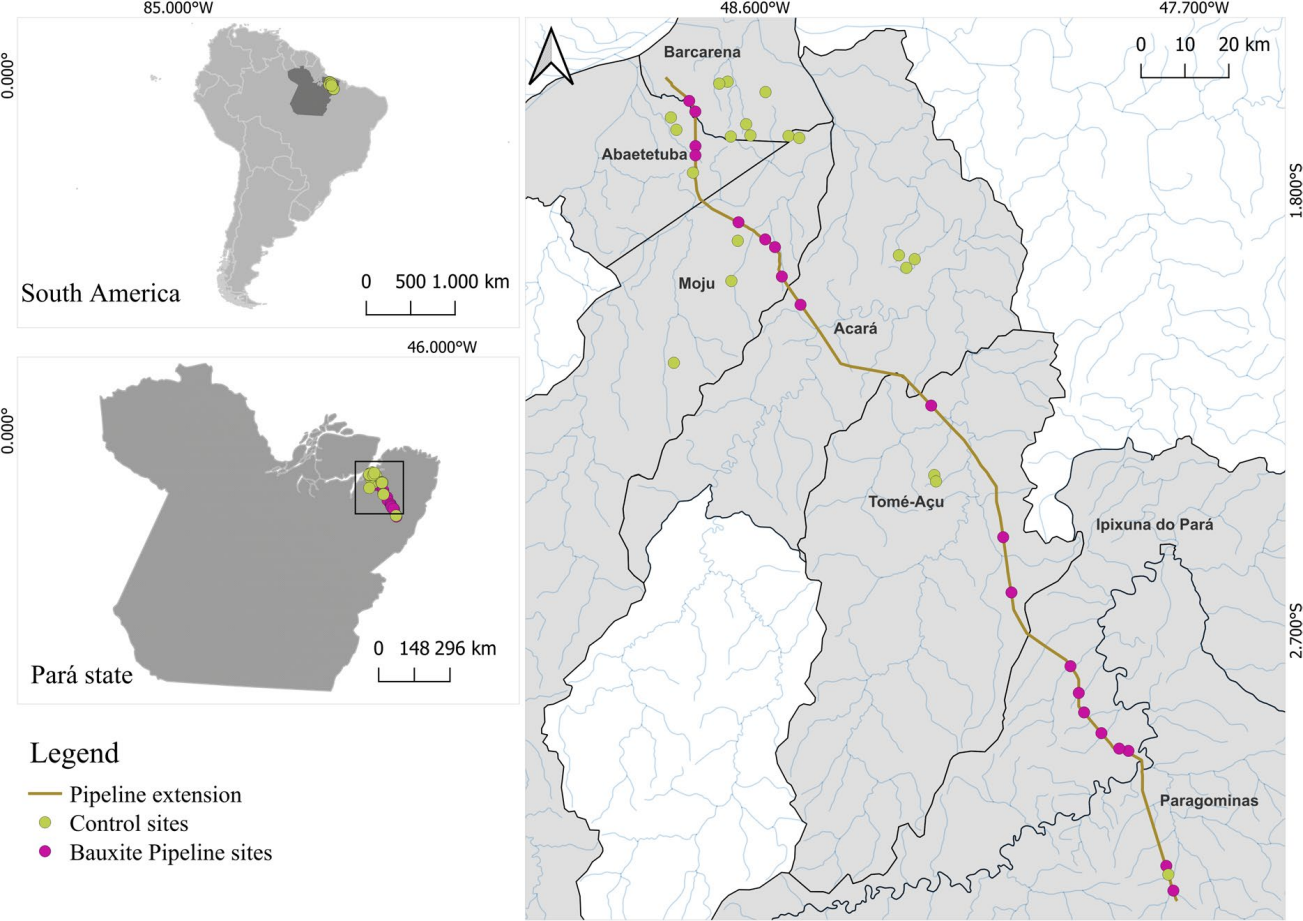
Location of the 40 sampled streams along the 244 km of the pipeline, Pará, Brazil. Green points represent control sites, while purple points indicate sites under the influence of the pipeline. The brown line represents the pipeline route

The study was conducted in the municipalities of Abaetetuba, Acará, Acará Mirim, Barcarena, Ipixuna, Moju, and Tomé-Açu in the state of Pará. The climate of the state is characterized by average temperatures between 22 °C and 28 °C, with relative humidity above 64% (Silva et al., 2021). The average annual rainfall ranges between 1000 mm and 3600 mm, with two well-defined rainy seasons: a more intense one from December to January and a drier one during the other months (Gama et al., 2007). The collections were carried out between 2022 and 2023, during the dry season, since this period presents greater biological and structural stability, allowing for a more consistent assessment of aquatic communities. A total of 40 streams were selected for the study, 20 located in the area of influence of the pipeline and 20 used as controls. The pipeline extends for 244 km, running close to several streams and to the beds of four major rivers (De Queiroz Lemos & Pimentel 2021; Othon et al., 2009) (Fig. 1).

### Slurry pipeline

The pipeline system, which extends for 244 km, consists of a steel tube with an external diameter of 24 inches and a thickness of 150 cm, capable of transporting approximately 9.9 million tons of bauxite slurry per year (Rodrigues et al., 2019). For its implementation, excavations were carried out along the route, allowing for the underground installation of the pipeline in order to avoid possible risks of accidents and leaks. In locations where excavation was not possible, bridges were built to enable the external passage of the pipeline over the streams. Roads were also constructed to carry out maintenance of the entire structure, many of which cross streams along the route.

### Sampling design and biological sampling

**Fig. 2:**
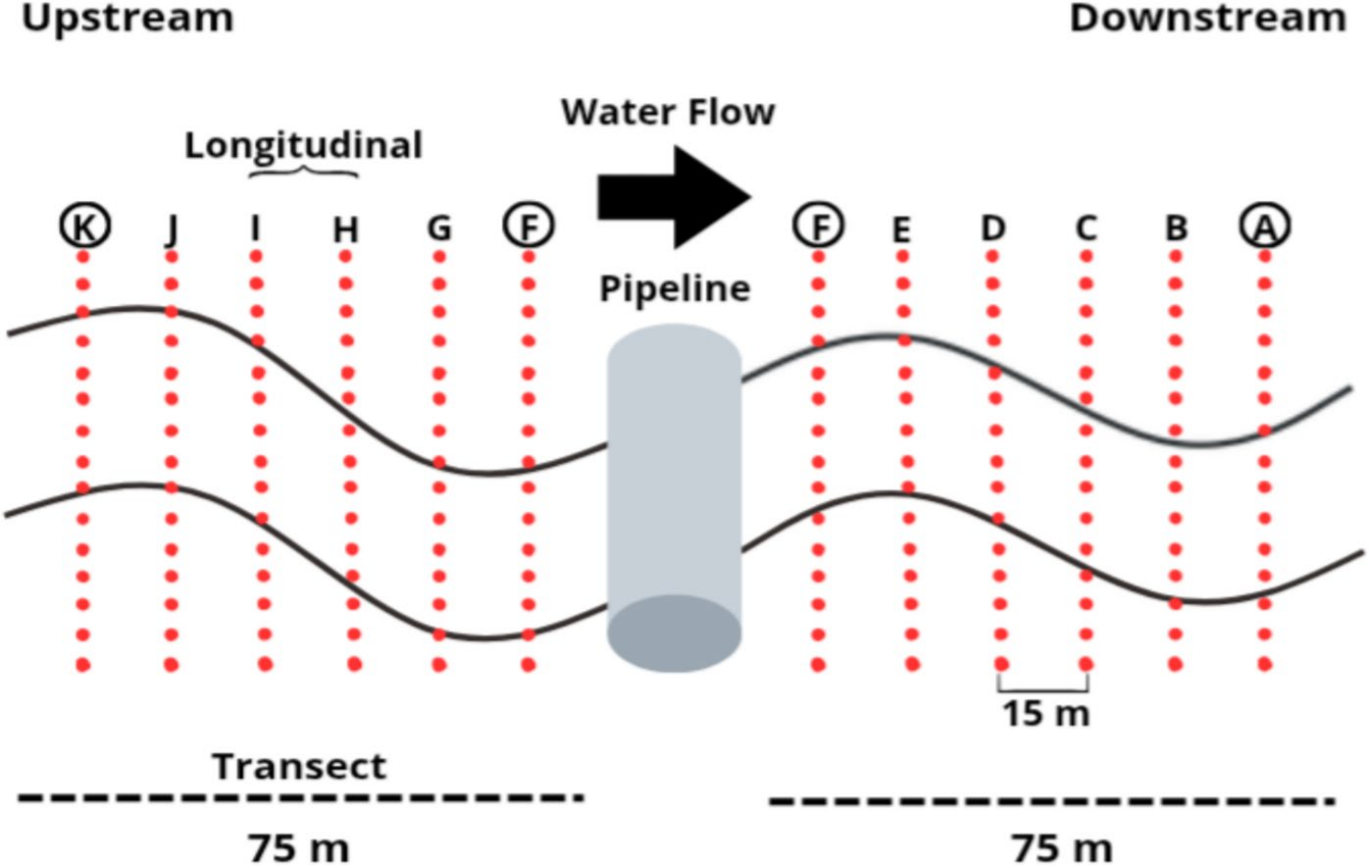
Sampling design carried out in pipeline streams showing the 150-m transect divided in half by the bridge with the pipeline passage

In each stream, a 150-m stretch was measured. In streams impacted by the pipeline, sampling was carried out in the upstream and downstream portions of the pipeline, covering 75 m in each section (Fig. 2). In control streams, samples were collected across 10 longitudinal sections of 15 m, distributed sequentially along the 150-m linear stretch, a division also applied in pipeline streams. Gerromorpha were actively collected on the water surface using a dip net with an 18-cm diameter and 1-mm mesh (Cunha et al., 2015). In each 15-m section, two samplings were conducted along the entire length of each 7.5-m subsection, resulting in two integrated samples per reach. The collected Gerromorpha were preserved in 85% ethyl alcohol and identified in the laboratory using dichotomous keys to the level of species or morphospecies (Kenaga, 1941; Kenaga, 1942; Nieser & Mello, 1997; Moreira et al., 2011; Moreira & Barbosa, 2014; Magalhães et al., 2016; Floriano et al., 2017).

Aiming to analyze the consequences of pipeline implementation in streams, distinguishing between upstream and downstream is essential in biomonitoring studies, as it helps identify patterns of human-induced disturbances (Sumudumali & Jaywardana, 2021). In areas affected by pipelines, downstream may receive greater loads of sediments and pollutants due to surface runoff and soil movement, which, together with the scarcity of riparian vegetation, can lead to changes in water quality and in the availability of essential microhabitats for aquatic macroinvertebrates (Silva et al., 2024). Comparing these two sections helps distinguish between natural and anthropogenic changes (Rosenberg & Resh, 1993), providing information for mitigation and biodiversity conservation in sensitive ecosystems such as Amazonian streams (Garcia et al., 2023).

### Habitat integrity index

To assess modifications in environmental integrity or to detect environmental changes, the habitat integrity index (HII) was applied to each sampled stream (Brazil et al., 2020a; Nessimian et al., 2008). This index consists of 12 questions that evaluate visual characteristics of the streams, and the questions are related to land use patterns in the riparian zone, riparian forest width, channel sediments, water flow, among others (Nessimian et al., 2008), generating a value for each stream ranging from 0 to 1, where 1 represents the most intact streams and 0 the most impacted.

### Environmental variables

Physical habitat variables were measured according to a simplified stream measurement protocol adapted from Peck et al. (2006) along each transect, while the water physicochemical variables were measured using a multiparameter probe. Five habitat variables considered important for the studied group were selected: riparian vegetation, estimated visually according to the protocol mentioned above (Peck et al., 2006); canopy cover, measured with a densiometer; HII; and dissolved oxygen (mg/L), measured with a Horiba U-50. These metrics were chosen because they represent important attributes of local stream conditions that can be affected by anthropogenic changes, in addition to being important predictors related to Gerromorpha diversity in previous studies (Cunha et al., 2015; Dias-Silva et al., 2020; Silva et al., 2024). Riparian vegetation is closely related to the distribution of species (Silva et al., 2024); canopy cover is important for the amount of light entering the stream, which affects water temperature (Cunha et al., 2015); the HII is considered a good predictor and exerts influence on Gerromorpha diversity (Brazil et al., 2020a, b); and dissolved oxygen can influence species richness indirectly by modulating habitat quality and community structure (da Silva et al., 2023; Croijmans et al., 2021).

### Statistical analysis

To test the first hypothesis, which aims to analyze the difference in species abundance and richness that will be greater between upstream and downstream sections of control streams compared to pipeline streams, the Shapiro–Wilk test (Shapiro & Wilk, 1965) was performed to test data normality, and the paired *T* test for independent samples (Gosset, 1908) was applied to evaluate the difference between each group. The second hypothesis, which aims to analyze if species abundance and richness will be greater in upstream sections than in downstream sections, in sites crossed by the pipeline, we performed the paired *T* test for dependent samples (Gosset, 1908).

For the third hypothesis, which aims to evaluate whether community composition differs between areas under the influence of the pipeline and control areas, we used permutational multivariate analysis of variance (PERMANOVA) to test the significance between groups with a Bray–Curtis distance matrix (Anderson, 2014). Indicator species were also calculated for each treatment using the indicator value method (IndVal; Dufrêne & Legendre, 1997). The indicator value is calculated based on comparisons of abundance and occurrence within species, without comparisons among species. This method assesses how well a species meets two criteria: specificity (occurrence restricted to a given treatment) and fidelity (frequency of occurrence within that treatment). Species with higher IndVal scores (%) are considered strong indicators because they have a greater probability of being recorded in that environment. In this study, species with IndVal values above 60% were designated as indicators of their respective habitats.

Finally, to test the fourth hypothesis, which investigates whether the pipeline structure has an indirect effect on the community, we used structural equation models (SEMs), which allow combining at least two linear models within the same analytical framework, enabling the testing of both direct and indirect effects among a set of variables of interest (Dos Santos et al., 2020; Wang et al., 2023). In our study, SEM was employed to relate habitat structure, environmental variables and local characteristics of the treatments with community abundance and richness (Supplementary Material 1). All analyses were carried out using the R software with the packages vegan and indicspecies (R Core Team, 2024).

## Results

A total of 2358 individuals were collected, belonging to 13 genera and 36 species across three families (Gerridae, Mesoveliidae, and Veliidae). The most abundant species were *Rhagovelia elegans* Uhler (1894) (*n* = 837), *Rhagovelia brunae* Magalhães et al., (2016) (*n* = 404), *Rhagovelia evidis* Bacon (1948) (*n* = 236), *Cylindrostethus palmaris* Drake and Harris (1934), and *Brachymetra lata* Shaw (1933), with 183 and 163 individuals, respectively (Supplementary material 2).

Species richness and individual abundance did not differ between the control and pipeline treatments, considering jointly the upstream and downstream portions of each stream (richness: *t* = 1.278, df = 36.91, *p* = 0.209; abundance: *t* = 1.515, df = 35.40, *p* = 0.139). In the second hypothesis, we verified that both richness (*p* = 0.210) and abundance (*p* = 0.317) did not differ between upstream and downstream in the control and pipeline treatments, which did not corroborate the first and second hypotheses.

**Fig. 3:**
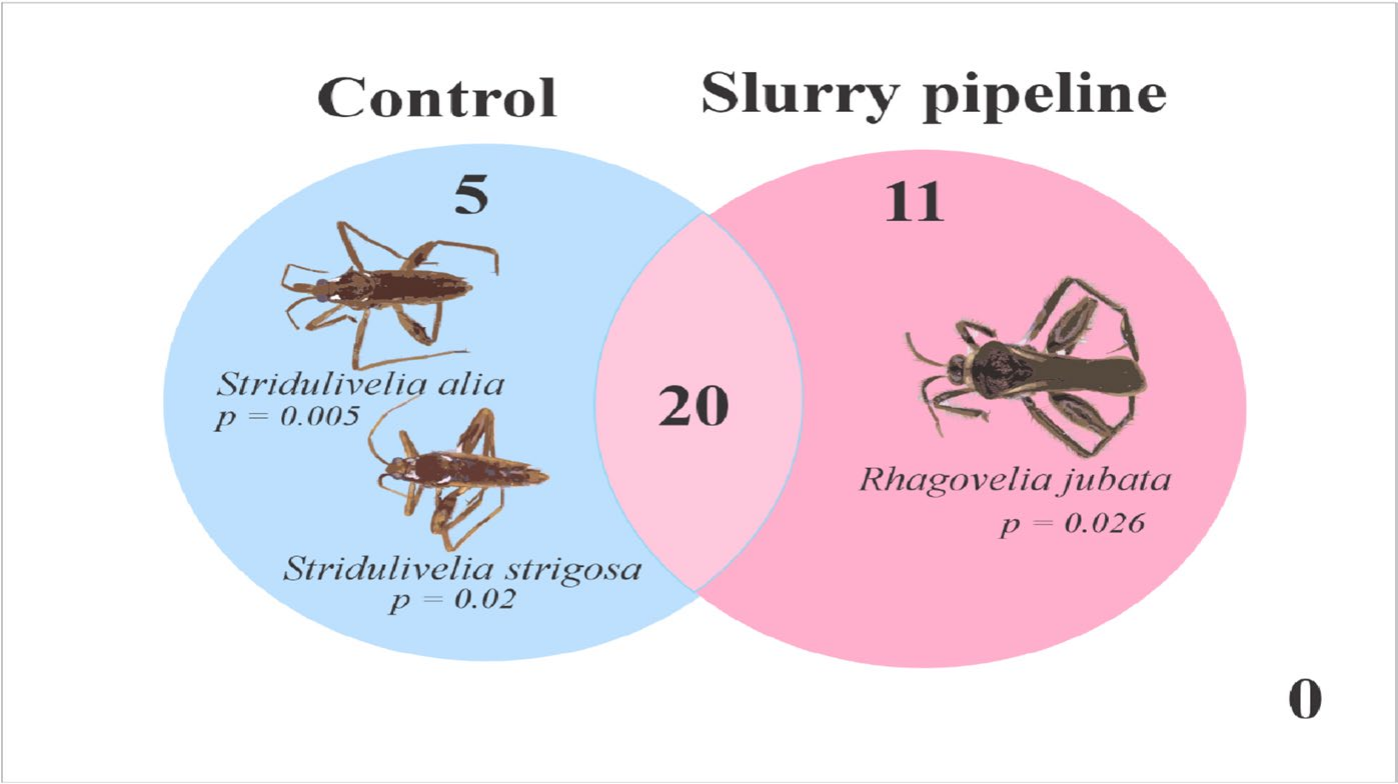
Venn diagram showing the species exclusive to the control and pipeline treatments, respectively

The species composition is significantly different between treatments (*F* = 2.11; *R*^2^ = 0.053; *p* = 0.013), corroborating our hypothesis (iii). Of the 36 species recorded in the study, 20 occur in both categories, 5 species occur exclusively in control streams, and 11 in the pipeline. IndVal identified three species with significant association to the treatments. The species *Stridulivelia alia* and *Stridulivelia strigosa* were indicative of the control treatment, while *Rhagovelia jubata* was indicative of the pipeline (Table 1 and Fig. 3). These results suggest that certain species have habitat preferences related to the presence of pipeline structures, which may reflect possible effects of local variables on the community.

**Table 1:** IndVal values for taxa associated with the control and pipeline groups. The column *index* indicates the association index used, and *p* shows the significance level of the association. Species with significant *p* are shown in bold

Group	Species	Index	IndVal	*p*
Control	*Euvelia discala*	1	0.387	0.216
	*Microvelia belterrensis*	1	0.224	1
	*Neogerris lotus*	1	0.396	0.310
	*Paravelia bullialata*	1	0.534	0.060
	***Stridulivelia alia***	1	0.654	0.005
	***Stridulivelia strigosa***	1	0.645	0.020
	*Stridulivelia tersa*	1	0.565	0.088
	*Stridulivelia transversa*	1	0.447	0.109
	*Tachygerris celosis*	1	0.335	0.629
Crossed by pipeline	*Callivelia conata*	2	0.224	1
	*Limnogonus recurvus*	2	0.303	0.726
	*Microvelia cf hormiga*	2	0.316	0.500
	*Microvelia cf venustatis*	2	0.224	1
	*Neogerris celeris*	2	0.224	1
	*Neogerris* sp.	2	0.224	1
	*Neogerris visendus*	2	0.224	1
	*Platyvelia brachialis*	2	0.224	1
	*Rhagovelia cf zecai*	2	0.316	0.492
	*Rhagovelia hambletoni*	2	0.316	0.483
	***Rhagovelia jubata***	2	0.548	0.026
	*Stridulivelia ayacucho*	2	0.224	1

**Fig. 4:**
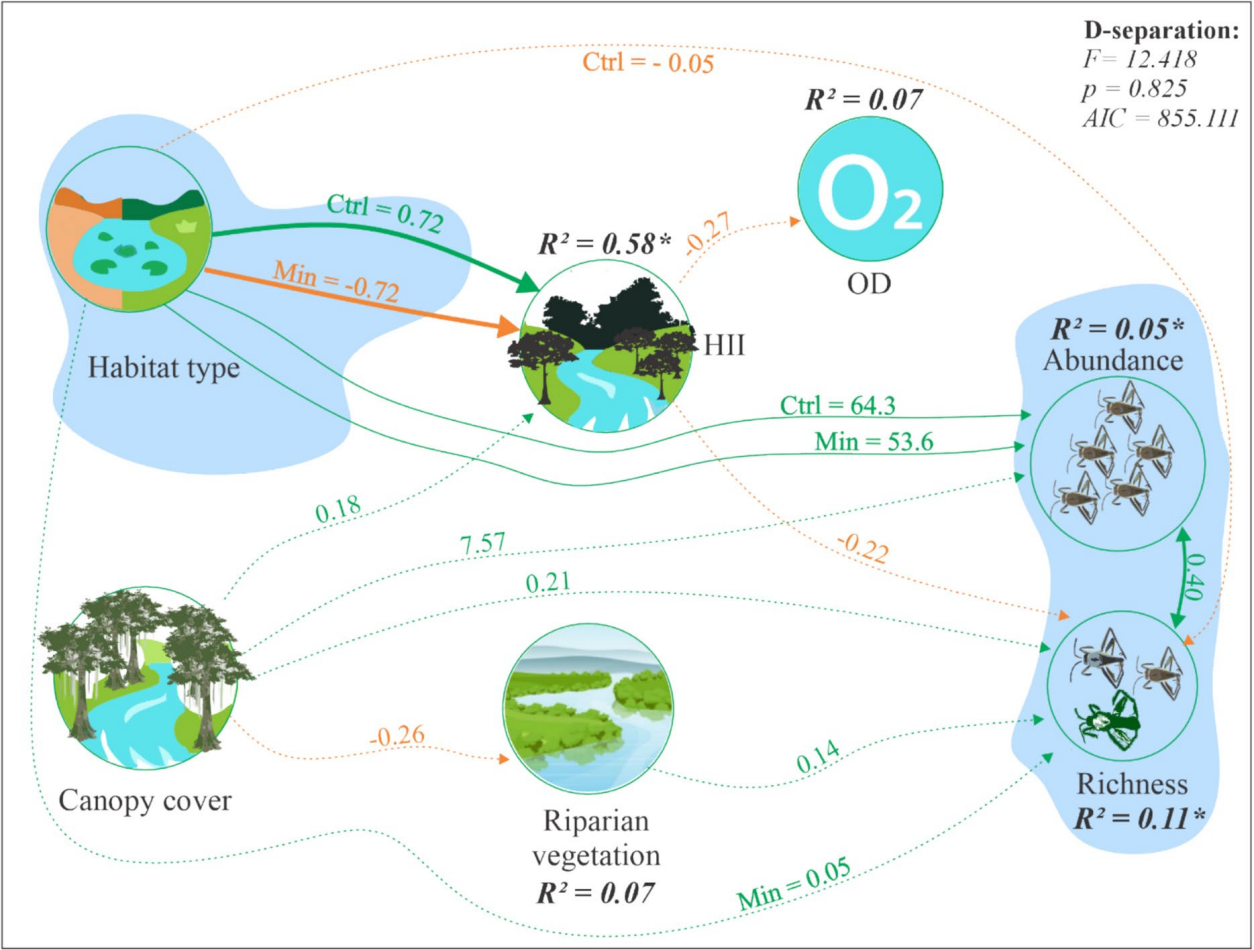
Structural equation model for habitat type (control and slurry pipeline) and local environmental variables for the semiaquatic bug community in Amazonian streams. OD—dissolved oxygen, HII—habitat integrity index, Ctrl—control, and Min—slurry pipeline. The thickness of the arrows represents the strength of the relationship (standardized *β*), with green = positive and orange = negative. Solid and dashed lines indicate significant and nonsignificant relationships, respectively. Only direct effects are shown. *p* values from D-separation greater than 0.05 indicate a good fit of the model to the data. The asterisk (*) represents the observed explanation for significant relationships

The structural equation model showed a good fit to the data (*F* = 12.418; *p* = 0.825; AIC = 855.111), indicating that the proposed relationships adequately explain the interactions between environmental variables and species abundance and richness. The HII was positively related in control environments (*β* = 0.72; *p* < 0.001) and negatively in pipeline environments (*β* = −0.72; *p* < 0.001), with 58% of the observed variance (*R*^2^ = 0.58). Abundance showed higher values in control areas (*β* = 64.30; *p* < 0.001) than in pipeline areas (*β* = 53.59; *p* < 0.001). Species richness was positively associated with abundance (*β* = 0.40; *p* = 0.005); however, its relationship with habitat type and HII was not significant (Fig. 4), which partially corroborated the fourth hypothesis.

## Discussion

In this study, we evaluated how the infrastructure associated with the installation and maintenance of a bauxite pipeline influences the community of semiaquatic bugs in streams of eastern Amazonia. Although species abundance and richness did not differ significantly between control and pipeline sites, our findings revealed detectable shifts in community composition, indicating that habitat structure plays an important role in shaping these assemblages (de Paiva et al., 2021; Lima et al., 2022). The indicator species analysis reinforced that *Rhagovelia jubata* was strongly associated with pipeline streams, whereas *Stridulivelia alia* and *Stridulivelia strigosa* characterized control environments. Together, these results suggest that even moderate alterations to riparian structure and local habitat conditions can influence community organization without necessarily reducing overall diversity.

By integrating multiple physical attributes such as vegetation cover, bank stability, presence of heterogeneous substrate, and the impact of human activities, the HII represented a robust indicator of habitat quality in the structural equation model (Brazil et al., 2020b; Nessimian et al., 2008). A large portion of the observed variance, regardless of the area type (control or crossed by pipeline), was explained by the HII, which has been associated with aquatic Gerromorpha as a tool for the conservation of Amazonian streams (Cunha et al., 2022; Freitas et al., 2025), reinforcing the importance of riparian vegetation and bank structure for Gerromorpha assemblages (Silva et al., 2024). In our study, the strong positive relationship between control streams and HII, as well as the negative relationship between pipeline-intersected streams and this index, demonstrates a loss of habitat quality resulting from the construction of infrastructure for pipeline installation. The bridges and roads built to allow vehicle access for pipeline maintenance were responsible for altering vegetation cover, one of the main factors considered by the index (Moy et al., 2022; Nessimian et al., 2008). Changes in local habitat conditions regulate community structure and the functional composition of aquatic insects (Nicacio et al., 2020).

Species composition differed between treatments, with pipeline streams showing a more heterogeneous species composition than control streams. This pattern is consistent with previous studies which establish that more intact streams allow greater dispersal of these organisms compared to streams altered by human activities (Guterres et al., 2021; Martins et al., 2024). IndVal revealed that *Stridulivelia alia* and *Stridulivelia strigosa* are specialists in environments without pipeline influence, whereas *Rhagovelia jubata* is associated with environments impacted by the pipeline. Previous research reported both *Stridulivelia alia* and *Rhagovelia jubata* as indicators of forested environments (Cunha & Juen, 2017). These contrasting patterns can be interpreted in light of differences in environmental context and the type and intensity of disturbance evaluated in each study (Ligeiro et al., 2013). In our study, the environmental changes caused by the pipeline may have exceeded the tolerance thresholds of *S. alia* but remained within the tolerance limits of *Rhagovelia jubata*, favoring its occurrence in impacted areas, due to the difference in niche occupied by the genera (Crumière et al., 2016). This suggests that although *Rhagovelia jubata* is generally associated with forested and shaded streams, its ecological plasticity allows it to persist in moderately altered environments (Santos-Silva et al., 2025). Conversely, *Stridulivelia alia* and *Stridulivelia strigosa* appear to be more sensitive to subtle habitat changes, reinforcing their role as indicators of well-preserved microhabitats (Santos et al., 2017).

Our findings revealed only subtle effects of the pipeline on the Gerromorpha community, reinforcing the established suitability of this group as a bioindicator in Amazonian streams (Cunha et al., 2015; Guterres et al., 2020; Dias-Silva et al., 2020). The limited magnitude of the impacts observed likely reflects the relatively moderate environmental alterations associated with the pipeline, which did not significantly affect species richness or abundance. Previous research has highlighted the central role of riparian vegetation in buffering disturbances and maintaining stream ecological integrity (Enríquez Espinosa et al., 2020; Silva et al., 2024), a pattern consistent with our study. Although changes in species composition provide relevant ecological signals, it is important to note that alternative ore-transport methods, such as truck-based systems, may cause more severe disturbances through increased sedimentation, vegetation removal, and pollutant emissions.

## Conclusion

Our results indicate that although the presence of the slurry pipeline did not significantly affect species richness or abundance, it did promote detectable shifts in community composition, reflecting habitat conditions. The presence of indicator species associated with each treatment underscores differences in habitat quality, particularly considering how riparian vegetation in pipeline areas appears to have helped maintain ecological stability and minimize disturbances. These results suggest that the environmental conditions in impacted streams may not have exceeded the tolerance limits of semiaquatic bugs, highlighting the need for management strategies that safeguard riparian vegetation and mitigate physical disturbances to maintain the functional integrity and biodiversity of these freshwater ecosystems.

Additional data for this study is available.

## Supplementary Information

Below is the link to the electronic supplementary material.

ESM 1 (PNG 706 KB)

ESM 2 (XLSX 11.9 KB)

## Data Availability

Not applicable.
