# Supplementary figures and images for: Effect of the mining pipeline on habitat quality and the diversity of semiaquatic bug communities (Heteroptera: Gerromorpha) in streams of the eastern Amazon
Source: Environ Monit Assess. 2026 Mar 5;198(3):276. doi: 10.1007/s10661-026-15086-7 (PMC12960337; doi:10.1007/s10661-026-15086-7)

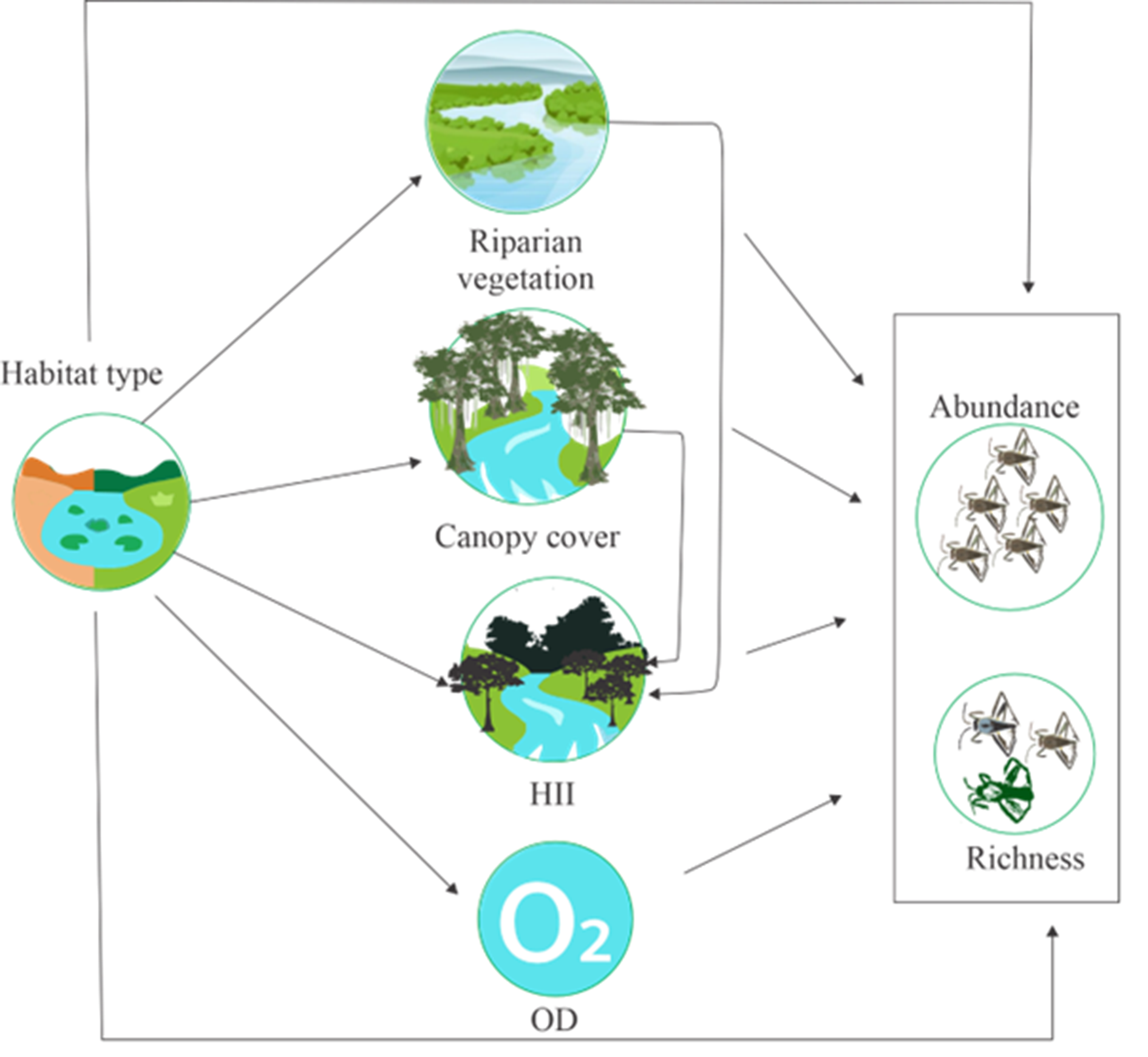

Supplement: Supplementary file 1 — (PNG 706 KB) [file 10661_2026_15086_MOESM1_ESM.png]
